# Comparison of Two Root Canal Filling Techniques: Obturation with Guttacore Carrier Based System and Obturation with Guttaflow2 Fluid Gutta-Percha

**DOI:** 10.3390/dj10040071

**Published:** 2022-04-15

**Authors:** Guido Migliau, Gaspare Palaia, Daniele Pergolini, Tommaso Guglielmelli, Roberta Fascetti, Afrah Sofan, Alessandro Del Vecchio, Umberto Romeo

**Affiliations:** Department of Oral and Maxillofacial Sciences, Sapienza University of Rome, Via Caserta 6, 00161 Rome, Italy; guido.migliau@uniroma1.it (G.M.); gaspare.palaia@uniroma1.it (G.P.); tommaso.guglielmelli@gmail.com (T.G.); roberta.fascetti@gmail.com (R.F.); afrahy28@yahoo.it (A.S.); alessandro.delvecchio@uniroma1.it (A.D.V.); umberto.romeo@uniroma1.it (U.R.)

**Keywords:** three-dimensional seal, marginal gaps, GuttaCore, GuttaFlow, microleakage

## Abstract

Introduction: The aim of the present study was to compare the quality of the root canal obturation obtained with two different techniques, i.e., thermoplastic gutta-percha introduced through a carrier (GuttaCore) and fluid gutta-percha (GuttaFlow2). Materials and Methods: The study included 40 permanent single-rooted human teeth, divided into two groups and obturated with Guttaflow (group G) and with GuttaCore (group T). The teeth were fixed and transversely sectioned, they were examined by scanning electron microscopy. The dentin–cement–gutta–percha interface and the percentage of voids produced by the two techniques were statistically analyzed. Results: GuttaCore showed a better filling in the apical third of the canal with a percentage of voids equal to 5%. GuttaFlow showed a lower percentage of voids in the middle and coronal thirds of the canal, 1.6% of coronal voids. Statistical analysis showed a statistically significant difference in the percentage of voids in the two groups (GuttaCore and Guttaflow2) in each portion. Conclusions: GuttaFlow2 seems to flow optimally in the middle and coronal third of the canal, with greater difficulty in filling the apical third. Due to the rigidity of the carrier, GuttaCore is able to reach better the most apical portions of the canals, with greater difficulty in creating the three-dimensional seal at the level of the middle third and coronal third.

## 1. Introduction

The formation of a three-dimensional seal is of fundamental importance in achieving long-term success following root canal therapy. Endodontic therapy includes several phases with specific goals. The first phase involves mechanical instrumentation and chemical irrigation of the root canal with the primary objective of eliminating infected tissue, microorganisms and their byproducts [1]. The next step is to perform obturation of the root canal system that leads to the formation of a three-dimensional and hermetic seal that is able to prevent any recontamination and also prevent periapical fluids to provide nourishment to microorganisms that survived cleansing and shaping procedures, in order to prevent possible multiplication [2]. In fact, it has been demonstrated that it is not possible to carry out complete cleaning and disinfection of the root canals because of the persistence of certain bacterial species [3]. The aim of endodontic treatment is therefore to reduce bacterial populations to levels compatible with healing [4], followed by filling the root canal system with a material capable of creating a three-dimensional seal in order to prevent bacterial micro-infiltration, the main cause of reinfection and failure of root canal treatment [5]. Gutta-percha is considered the gold standard material for root canal fillings [6]. Due to its superior properties, it allows a significantly lower percentage of voids (1.02%) [7] compared to previously used materials. Through this study where we compare two different root canal filling techniques we want to evaluate if one can be considered better than the other. One of the most recent techniques involves filling the root canal with “GuttaFlow2” fluid gutta-percha, using a cold filling technique. “GuttaFlow2^®^” (Coltene/Whaledent, Altstatten, Switzerland) is a combination of gutta-percha powder, poly-dimethylsiloxane, and silver particles. Its ability to expand slightly and its higher flowability allow good adaptation to the root canal walls, with an average marginal gap of 2.38 ± 1.43 μm [8] and good adaptation at the level of the apical third [9]. Moreover, several studies have demonstrated the high biocompatibility and lower cytotoxicity of GuttaFlow2 [10,11] compared to other root canal sealants (GP). Despite its homogeneous composition, it produces a certain level of surface porosity [12]. Another filling method involves the use of core carriers coated with thermoplastic gutta-percha. The carriers examined so far are cores made of polysulfone plastic material (Thermafil Plus) or of Vectra, a liquid crystal polymer (Thermafil). The studies carried out on these systems have shown their effectiveness in performing three-dimensional fillings of the root canal, with higher volumes of obturation of the lateral canals compared to other techniques of hot obturation, in a shorter period of time than the lateral condensation technique [2,13]. A new core-carrier system is the GuttaCore, whose core is made of cross-linked thermoplastic gutta-percha (GuttaCore, Dentsply Tulsa Dental Specialties, Tulsa, OK, USA). Previous studies have shown how these carriers allow quicker and easier removal of the material from the root canal in case of retreatment [14]; however, there are not enough studies in the literature to demonstrate the quality of the root canal obturation achieved with this core-carrier system. The aim of the present study is to analyze the three-dimensional seal obtained with the new core-carrier system made of thermoplastic cross-linked gutta-percha (GuttaCore), compared to Guttaflow2. The study assesses the filling capacity using scanning electron microscopy and analyze the percentage of voids of the GuttaCore system and compare it with the GuttaFlow2 system. Therefore, by calculating the percentage of voids and the quality of root canal filling at the level of the apical, middle, and coronal third, and determining if one method is better than the other.

## 2. Materials and Methods

### 2.1. Preparation and Filling of Root Canals

The study included 40 single-rooted lower premolar permanent human teeth extracted for periodontal or orthodontic reasons. Root length standardized to 13 mm. Teeth with cracks, resorption, calcification, or open apex were excluded. Three different operators were selected, and with the toss of the coin we randomized a technique to be performed to each operator. Standard access cavities were made: a k-file of size 10 (Maillefer) was introduced into each canal; the working length was determined by periapical radiography using the parallel ray technique, thus avoiding deformations and allowing precise measurements. Each individual tooth was checked radiographically for correspondence between the tip of the file introduced into the canal and the root apex, thus determining the working length. We proceeded with the instrumentation of the canals: the coronal third of each canal was countersunk using a Largo bur (Largo, Dentsply, St Quentin en Yvelines, France) of sizes 4, 3, 2, and 1. The root canals were then prepared using hyflex CM nickel-titanium (NiTi) (coltene-Waledent, Allstetten, Switzerland) files, cleaning and shaping the root canal by crown-down technique using the sequence of files: 08/25, 06/20, 04/20 reaching the apex; we then proceeded to the final preparation with 04/25, 04/30, 04/35.

Canals were irrigated with 2 mL of 5% NaOCl after each instrument change. Apical patency was maintained by introducing a 10 k file 1 mm beyond the working length after each instrument. Measurement of the circumference of the apical foramen was performed using Ni-Ti k-file with 0.2 taper. According to the measurement, the apical portion was increased to a maximum of 0.35 mm or 0.40 mm (with hyflex 06/35-06/40). After completion of the preparation, all samples were dried with sterile paper tips (Dentsply Maillefer, Tulsa, OK, USA). Once the root canal preparation was completed, the teeth were randomly divided into two experimental groups: T, GuttaCore, and G, GuttaFlow2.

Group T: GuttaCore Obturator (Dentsply Tulsa Dental Specialties). A verifier was selected that would passively fit the canal, and then the corresponding size obturator (size 35 or 40) was selected. A thin layer of sealant (Argoseal, Milton Keynes, UK) was placed along the walls of the entire canal, the GuttaCore obturator was then heated in the oven for 30 s (in accordance with the manufacturer’s recommendations). The heated GuttaCore obturator was then slowly inserted into the canal at the previously established working length, without twisting or forcing it.

Group G: The filling of the root canal was carried out according to the manufacturer’s instructions. A small amount of GuttaFlow2 was placed into the canal; next, the main cone of GuttaPercha, of the size corresponding to that of the last file used for the instrumentation of the canal, was slowly inserted into the canal until it reached the previously determined working length. It was then slowly retracted and twisted back, ensuring the complete wetting of the cone and of the canal walls.

**Figure 1:**
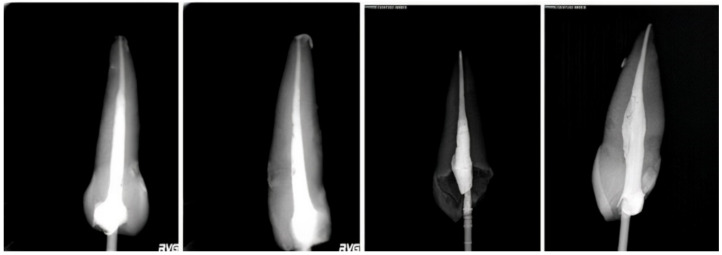
Rx GuttaFlow, Rx GuttaCore, Rx buccal, Rx mesial respectively.

Periapical radiographs with mesial and buccal projection were taken for each element in order to obtain a complete evaluation of the examined specimens (Figure 1).

Statistical analysis was then performed using Graphpad Prism software. Data were analyzed by Student’s *t*-test (one-way analysis of variance, ANOVA), with statistical significance set at *p* < 0.05.

### 2.2. Preparation of Scanning Electron Microscopy

**Figure 2:**
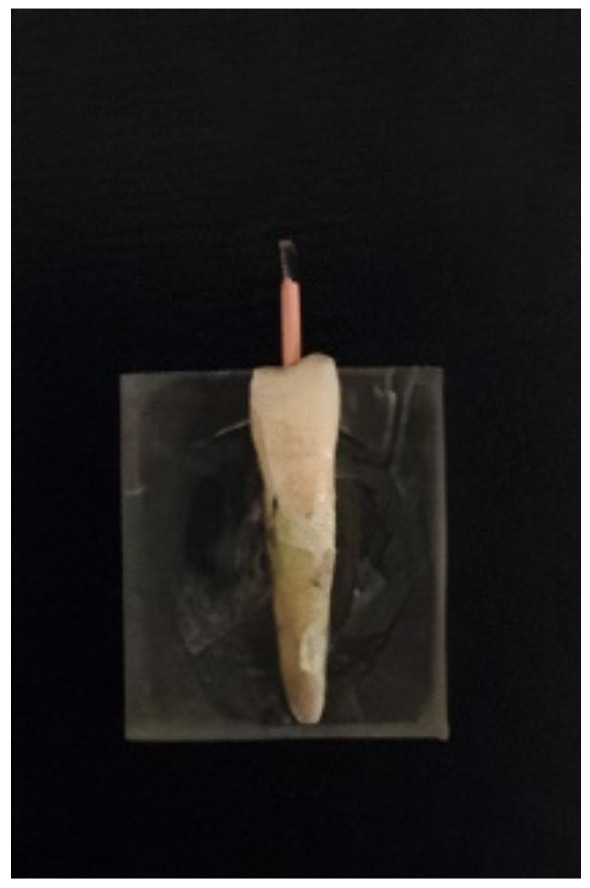
Tooth fixed on block.

The teeth were fixed on a polycarbonate block with cyanoacrylate-based glue to secure them during cutting (Figure 2) and were then transversely sectioned at 3, 6, 9, and 12 mm from the apex.

The sections were made perpendicular to the longitudinal axis of the root. A first cut was made, using a 0.3 mm diamond blade (Micromet SECOTRON 200, Svogerslev, Denmark) at 1500 rpm under continuous cooling with water jet creating a notch on the tooth surface.

The final section was obtained by means of a blade and hammer, causing complete fracture of the tooth. This type of fracture made it possible to reduce the formation of debris that is normally generated during cutting.

In order to flatten the sample, each surface to be examined was polished with sandpaper (4000 grit SiC paper) a single grain size was used to minimize artifacts and finally treated with an application of 17% EDTA for 18 min under ultrasound was made to obtain a shiny surface to be examined with the scanning electron microscope (SEM).

This removed the smear layer exposing the surface. Finally, the polished surface was rinsed with deionized water.

In order to evaluate the interaction between the samples and the electron beam, the samples must be conductive. Therefore, they were mounted on metal supports and treated with chromium. Finally, the samples were examined using a scanning electron microscope (Zeiss, Auriga, Germany), at 1000× magnification.

**Figure 3:**
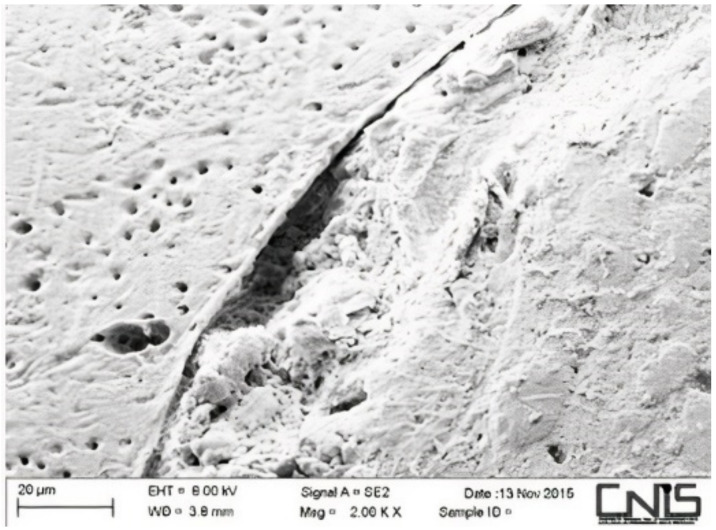
Interface between dentin and sealant. Section of third apical third T system.

**Figure 4:**
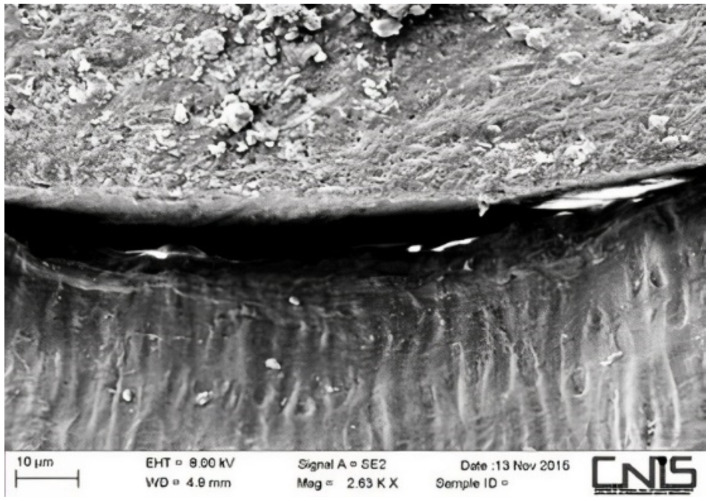
Interface between dentin and sealant. Section of medium third T system.

**Figure 5:**
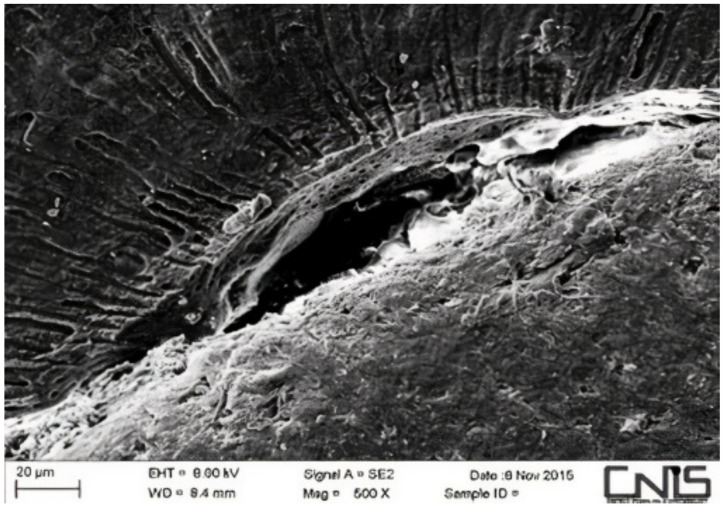
Interface between dentin and sealant. Section of second medium third T.

**Figure 6:**
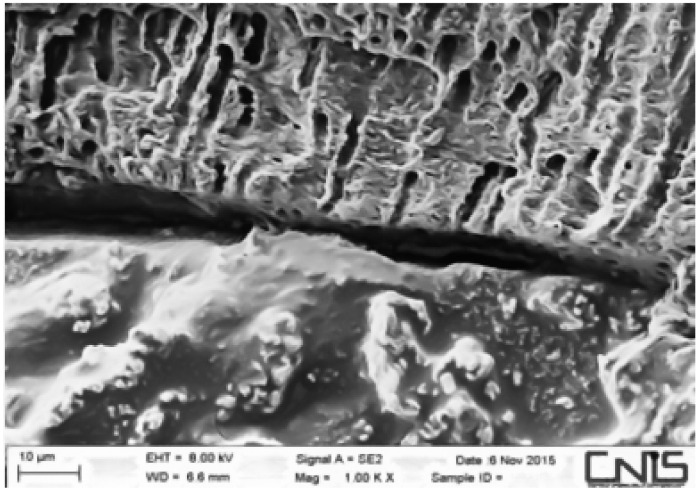
Interface between dentin and sealant. Section of coronal third T system.

**Figure 7:**
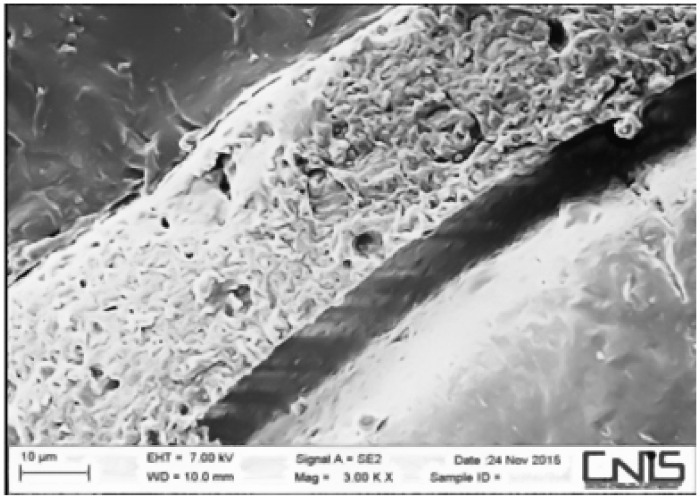
Interface between dentin and sealant. Section of apical third G system.

**Figure 8:**
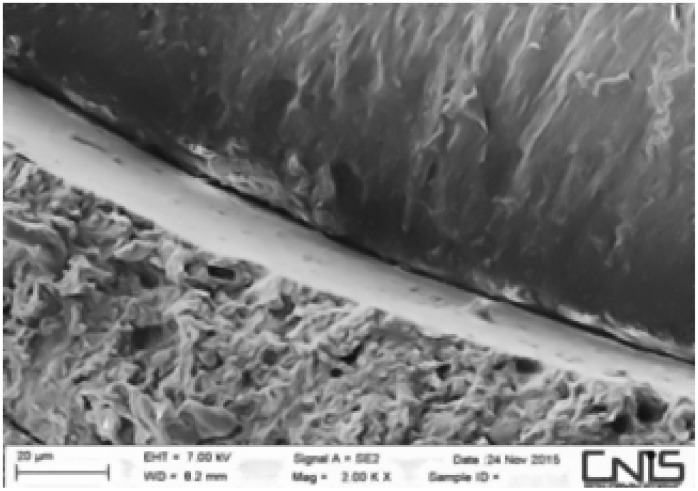
Interface between dentin and sealant. Section of medium third G system.

**Figure 9:**
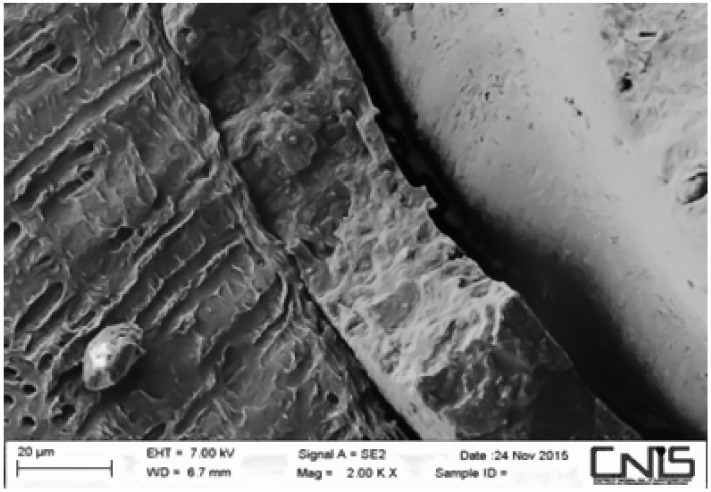
Interface between dentin and sealant. Section of the second medium third G system.

**Figure 10:**
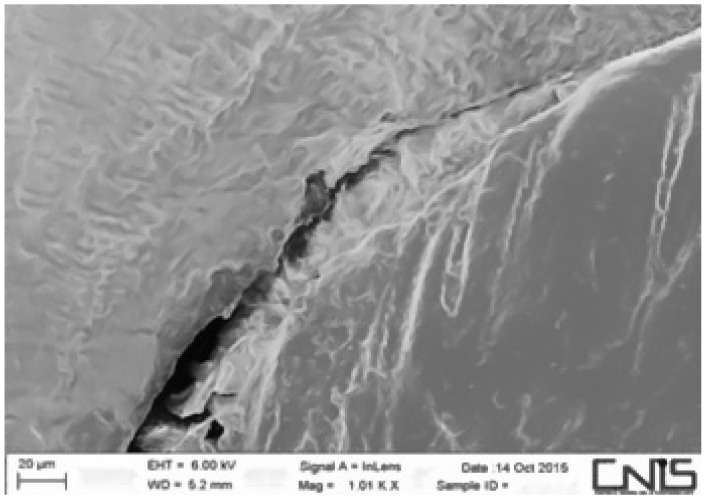
Interface between dentin and sealant. Section of coronal third G system.

Observations were made at the dentin–cement–gutta–percha interface (Figure 2, Figure 3, Figure 4, Figure 5, Figure 6, Figure 7, Figure 8, Figure 9 and Figure 10). The digital images were processed with the ImageJ program (Rasband WS, ImageJ; US National Institute of Health, Bethesda, MD, USA), measuring the gutta-percha, the sealant and the voids present (Figure 2, Figure 3, Figure 4, Figure 5, Figure 6, Figure 7, Figure 8, Figure 9 and Figure 10), procedure that automatically calculates the software. The minimum size of the voids considered is 0.7 micron as this is a good approximation of the minimum space in which biofilm formation can occur. The measurement of the voids was carried out using the software “analyze particles”. To obtain the percentage of voids present, the linear dimensions of the voids were compared with the perimeter of the dentin–sealant interface. By analyzing the percentages thus obtained for each section, we are able to compare the two materials and to have a 3D statistic of the voids produced by the two methods.

Statistical analysis was then performed using Graphpad Prism software. Data were analyzed by Student’s *t*-test (one-way analysis of variance, ANOVA), with statistical significance at *p* < 0.05.

## 3. Results

**Figure 11:**
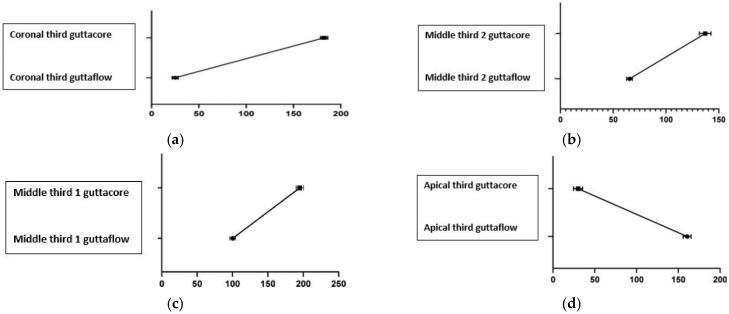
(**a**) Percentage (%) of voids in the coronal third: 12.1% group T–1.6% group G; (**b**) Percentage (%) of voids in the middle third 2: 20.5% group T–6.8% group G; (**c**) Percentage (%) of voids in the middle third 1: 17.25% group T–12.5% group G; (**d**) Percentage (%) of voids in the apical third: 5% group T–26% group G.

The results of this pilot study highlight the percentage of voids present at the interface between the material used in filling the canal and the dentinal wall. They were analyzed by subdividing the teeth into four portions (Figure 11a–d): coronal portion, middle 1, middle 2, apical third, through cross-sections at 3, 6, 9, and 12 mm from the apex.

The analysis of the results shows that:

- The GuttaCore System shows a better filling at the level of the apical third with a percentage of voids equal to 5%. The percentage of voids increases at the level of the coronal third and middle third of the root canal equal to: 12.1% coronal voids; 20.5% in the middle portion 2; 17.25% in the middle portion 1 (Table 1).
- On the contrary, because of its greater fluidity, the GuttaFlow System allows a better filling at the coronal level and in the middle third, with a percentage of voids equal to 1.6% coronal; 6.8% in the middle portion 2; 12.5% in the middle portion 1; on the contrary, the percentage of voids at the apical interface increased significantly, equal to 26% (Table 1).

The difference in the percentage of voids in the two groups (GuttaCore and Guttaflow2) was statistically significant at all levels, coronal third, middle third, and apical third (Figure 11a–d). In particular, in the coronal third, there is a *p* value < 0.0001; in the middle third 2 *p* < 0.0001; middle third 1 *p* < 0.0001; and apical third *p* < 0.0001.

The results of the statistical analysis were then represented in Table 1 and in four graphs, by which it is possible to observe an inversion of the trend passing from the coronal third to the apical third. The gaps present at the coronal level (Figure 11a) increase in the GuttaCore technique compared to the Guttaflow2 technique; in the middle third 2 and middle third 1 (Figure 11b,c), the difference between the two methods reduces, retaining, however, a higher percentage for the GuttaCore technique; in the fourth graph (apical portion) (Figure 11d), we see an inverse trend due to the fact that the percentage of gaps is higher with GuttaCore than with GuttaFlow2.

## 4. Discussion

The main cause of endodontic failure is the persistence of microorganisms capable of causing intraradicular or extraradicular infection [15]. Therefore, it is desirable to perform an excellent cleaning and shaping of the root canal combined with an obturation technique that provides a three-dimensional closure of the system, minimizing the formation of gaps and voids, a possible source of communication with the outside and, therefore, reinfection and failure of the treatment itself [16]. Previous endodontics studies have shown excellent physical and chemical qualities of the Guttaflow2 material, demonstrating how, because of its good adaptation to the canal walls, it is possible to obtain a filling equal to 99.72%, in terms of total filling. Through this study, it was possible to analyze the difficulties of the material in penetrating the apical third, leading to an increase in the gaps present between the root canal wall and the sealant and an increase in the amount of sealant, to the detriment of the percentage of gutta-percha inserted. On the contrary, the GuttaCore system showed a better filling of the apical third, with a much lower percentage of gaps (Table 1); the interfacial gaps increased instead at the level of the middle third and the coronal third (Figure 11a–c).

The limitations of this study were two: the first did not select a statistically significant number of samples and the second was that it was decided to dissect the tooth by fracture, with the risk of altering the gap between gutta-percha and tooth.

In this study, GuttaFlow2 was used in the Single Cone Cold Filling Technique because of the excellent physical and chemical qualities of the material: low solubility with good stability over time [17], expansiveness improving the sealing capacity over time [18], and fluidity and smoothness.

Root canal filling was performed according to the manufacturer’s instructions. After introducing a minimal amount of fluid gutta-percha, the main cone was slowly inserted into the canal, and then slowly pulled and twisted back and forth to ensure complete wetting of the cone and canal wall. Previous studies have shown that GuttaFlow, used in the single-cone technique, has good adaptation to the canal walls [19], due in part to its ability to expand following its insertion [20]. It has been demonstrated that the filling percentage of GuttaFlow2, in terms of total filling, is 99.72% [21]; at the level of the apical third of the root canal system, the formation of marginal gaps equals to 3.51 ± 1.81 μm has been reported [9].

This study shows, in agreement with the data present in the literature, that the GuttaFlow2 system has a higher percentage of voids in the apical third (Table 1); moreover, it is in fact difficult to control the penetration of the material in the narrow space of the apical third with this system. Moreover, in the single cone technique, the gutta-percha master cone is not compacted but is inserted at working length with a considerable amount of sealant. This does not promote the penetration of the cone into the apical portion, leading to a high volume of sealant. All this promotes the formation of voids and reduces the sealing quality of the filling itself [22]. In fact, it has been observed that sealants do not form a continuous layer between the gutta-percha and the canal wall, thus creating gaps at the gutta–percha–dentin interface [23]. The middle third and the coronal third, on the other hand, present wider sections, allowing a more complete and efficient filling due to the high fluidity of GuttaFlow2.

The second technique investigated in this study involves the use of GuttaCore. Previous studies have analyzed the quality of the obturation obtained through the use of systems based on plastic carriers (Thermafil). The results of these studies show that these systems produce a higher percentage of gutta-percha compared to other obturation techniques (System B, cold lateral condensation) [24], with reduced working times compared to the lateral condensation technique [25]. On the other hand, there is little information in the literature about carriers made of semi-rigid thermostable gutta-percha, such as the GuttaCore System. Among the most recent studies, the one conducted by Ruth Pérez-Alfayate et al. shows that these carriers, in particular the GuttaFusion and GuttaCore Systems, produce very homogeneous fillings, with a high percentage of areas filled with GuttaPercha and a low percentage of voids, respectively, at the coronal (GuttaFusion) and apical (GuttaCore) levels [26,27,28].

The strength of this study is that it has been shown how the thermoplastic gutta-percha carrier condenses effectively in the apical third (Table 1): its low viscosity and high thixotropy allow the material to flow at working length, effectively filling the apical third; moreover, towards the apex, the section of the canal is round and this allows a better penetration of gutta-percha. On the contrary, a higher percentage of voids was found in the middle and coronal third (Figure 11a–c); the cause is due to the anatomical complexity of the canal system. Moreover, in the different morphology between the gutta-percha carrier and the root canal: one with a circular section and the other with an oval section, therefore with the difficulty of sealing the entire surface [29,30,31,32].

## 5. Conclusions

Based on the results of the present study, it can be concluded that GuttaFlow2 is able to flow optimally in the middle and coronal third of the canal (Table 1), even in canals with more complex anatomies. Despite its high fluidity, however, difficulties were encountered in reaching the more apical portions of the canal. In fact, the tip mounted on the syringe, which distributes the cement inside the canal, cannot reach the portions near the root apex, thus increasing the percentage of voids between the sealant and the canal wall in this area (Figure 11d). Moreover, the introduction of the single cone of gutta-percha does not foresee the vertical compaction, which leads to a difficult penetration of the cone in the narrow apical space, resulting in an increase in the amount of sealant with an increase in the gaps [33,34,35,36].

On the contrary, the Guttacore System is able to penetrate optimally into the apical third of the canal, thus reducing the number of voids at this level (Table 1). In fact, the carrier, being rigid, can reach the working length, thus obtaining an excellent apical seal (Figure 11d). The number of voids, on the other hand, increases in the middle third and the coronal third (Table 1), probably due to the discrepancy between the morphology of the carrier, with its round cross-section, and the morphology of the root canals, with a more oval cross-section [37,38,39,40].

In conclusion, it can be said that both systems have shown excellent filling qualities and in order to make the most of their advantages and minimize their limitations, a combination of the two techniques might be considered. However, this remains to be tested [41,42].

## Figures and Tables

**Table 1:** Results table. the results show the values for group G and group T, respectively, in terms of the void dimensions, mean value, and percentage value in their respective four points analyzed: coronal, mean 2 mean 1 and apical.

Group G (Guttaflow)				Group T (Guttacore)			
	Width + Height (µm)	Mean	Void %		Width + Height (µm)	Mean	Void %
Coronal	49.86	24.92	1.6	Coronal	364.87	182.43	12.1
Middle 2	130.84	65.42	6.8	Middle 2	390.27	195.13	20.5
Middle 1	200.76	100.38	12.5	Middle 1	274.19	137.09	17.25
Apical	321.27	160.06	26	Apical	60.05	30.02	5

## Data Availability

The data shown are at the complete disposal of the scien-tific community and can be found through the corre-sponding author daniele.pergolini@uniroma1.it.

## References

[1] Junior J.F.S., Rôças I.d.N., Marceliano-Alves M.F., Pérez A.R., Ricucci D. (2018). Unprepared root canal surface areas: Causes, clinical implications, and therapeutic strategies. Braz. Oral. Res..

[2] Greco K., Cantatore G. (2014). A critical approach to the root canal obturation techniques. Giornale Ital. Di Endod..

[3] Sakamoto M., Siqueira J.F., Rôças I.N., Benno Y. (2007). Bacterial reduction and persistence after endodontic treatment procedures. Oral. Microbiol. Immunol..

[4] Siqueira J.F., Rôças I.N. (2008). Clinical implications and microbiology of bacterial persistence after treatment procedures. J. Endod..

[5] Muliyar S., Shameem K.A., Thankachan R.P., Francis P.G., Jayapalan C.S., Hafiz K.A.A. (2014). Microleakage in Endodontics. J. Int. Oral. Health.

[6] Vishwanath V., Rao H.M. (2019). Gutta-percha in endodontics—A comprehensive review of material science. J. Conserv. Dent..

[7] Hammad M., Qualtrough A., Silikas N. (2009). Evaluation of Root Canal Obturation: A Three-dimensional In Vitro Study. J. Endod..

[8] Jain S., Adhikari H.D. (2018). Scanning electron microscopic evaluation of marginal adaptation of AH-plus, GuttaFlow, and RealSeal at apical one-third of root canals—Part I: Dentin-sealer interface. J. Conserv. Dent..

[9] Adhikari H.D., Jain S. (2018). Scanning electron microscopic evaluation of marginal adaptation of AH-Plus, GuttaFlow, and RealSeal at apical one-third of root canals—Part II: Core-sealer interface. J. Conserv. Dent..

[10] Collado-González M., Tomás-Catalá J.C., Oñate-Sánchez R.E., Moraleda J.M., Rodríguez-Lozano F.J. (2017). Cytotoxicity of GuttaFlow Bioseal, GuttaFlow2, MTA Fillapex, and AH Plus on Human Periodontal Ligament Stem Cells. J. Endod..

[11] Accardo C., Himel V.T., Lallier T.E. (2014). A Novel GuttaFlow Sealer Supports Cell Survival and Attachment. J. Endod..

[12] Uyanik M.O., Nagas E., Cubukcu H.E., Dagli F., Cehreli Z.C. (2010). Surface porosity of hand-mixed, syringe-mixed and encapsulated set endodontic sealers. Oral. Surg. Oral. Med. Oral. Pathol. Oral. Radiol. Endod..

[13] Suguro H., Takeichi O., Hayash M., Okamura T., Hira A., Hirano Y., Ogiso B. (2018). Microcomputed tomographic evaluation of techniques for warm gutta-percha obturation. J. Oral. Sci..

[14] Faus-Llácer V., Pérez R.L., Faus-Matoses I., Ruiz-Sánchez C., Zubizarreta-Macho Á., Sauro S., Faus-Matoses V. (2021). Efficacy of Removing Thermafil and GuttaCore from Straight Root Canal Systems Using a Novel Non-Surgical Root Canal Re-Treatment System: A Micro-Computed Tomography Analysis. J. Clin. Med..

[15] Prada I., Micó-Muñoz P., Giner-Lluesma T., Micó-Martínez P., Collado-Castellano N., Manzano-Saiz A. (2019). Influence of microbiology on endodontic failure. Literature review. Med. Oral. Patol. Oral. Cir. Bucal..

[16] Tabassum S., Khan F.R. (2016). Failure of endodontic treatment: The usual suspects. Eur. J. Dent..

[17] Zhou H., Shen Y., Zheng W., Li L., Zheng Y., Haapasalo M. (2013). Physical properties of 5 root canal sealers. J. Endod..

[18] Kontakiotis E.G., Tzanetakis G.N., Loizides A.L. (2007). A l2-month longitudinal in vitro leakage study on a new silicon-based root canal filling material (Gutta-Flow). Oral. Surg. Oral. Med. Oral. Pathol. Oral. Radiol. Endod..

[19] Zielinski T.M., Baumgartner J.C., Marshall J.G. (2008). An evaluation of Guttaflow and gutta-percha in the filling of lateral grooves and depressions. J. Endod..

[20] Ørstavik D., Nordahl I., Tibballs J.E. (2001). Dimensional change following setting of root canal sealer materials. Dent. Mater..

[21] Mohammad Y., Alafif H., Hajeer M.Y., Yassin O., Patil S. (2016). An Evaluation of GuttaFlow2 in Filling Artificial Internal Resorption Cavities: An in vitro Study. J. Contemp. Dent. Pr..

[22] Wu M.K., Kast’áková A., Wesselink P.R. (2001). Quality of cold and warm gutta-percha fillings in oval canals in mandibular premolars. Int. Endod. J..

[23] Machado M.E.d., Shin R.C.F., Zólio A.A., Pallotta R.C., Nabeshima C.K. (2010). Confronto Tra La Quantità di Sigillante Nell’Otturazione Canalare Con L’uso di Strumentazione e tecniche D’otturazione Diverse. Il Dent Mod.

[24] Gençoğlu N. (2003). Comparison of 6 different gutta-percha techniques (part II): Thermafil, JS Quick-Fill, Soft Core, Microseal, System B, and lateral condensation. Oral Surg. Oral. Med. Oral. Pathol. Oral Radiol. Endodontol..

[25] Mohan S.M., Kaushik S. (2009). Root Canal Treatment Using Thermoplasticized Carrier Condensation Technique. Med J. Armed Forces India.

[26] Pérez-Alfayate R., Mercade M., Algar-Pinilla J., Cisneros-Cabello R., Foschi F., Cohen S. (2021). Root Canal Filling Quality Comparison of a Premixed Calcium Silicate Endodontic Sealer and Different Carrier-Based Obturation Systems. J. Clin. Med..

[27] Kato H., Nakagawa K.-I. (2010). FP core carrier technique: Thermoplasticized gutta-percha root canal obturation technique using polypropylene core. Bull. Tokyo Dent. Coll..

[28] Kumar N.S.M., Prabu P.S., Prabu N., Rathinasamy S. (2012). Sealing ability of lateral condensation, thermoplasticized gutta-percha and flowable gutta-percha obturation techniques: A comparative in vitro study. J. Pharm. Bioallied. Sci..

[29] Neuhaus K.W., Schick A., Lussi A. (2016). Apical filling characteristics of carrier-based techniques vs. single cone technique in curved root canals. Clin. Oral. Investig..

[30] Schäfer E., Nelius B., Bürklein S. (2012). A comparative evaluation of gutta-percha filled areas in curved root canals obturated with different techniques. Clin. Oral. Investig..

[31] Li G.-H., Niu L.-N., Selem L.C., Eid A.A., Bergeron B.E., Chen J.-H., Pashley D.H., Tay F.R. (2014). Quality of obturation achieved by an endodontic core-carrier system with crosslinked gutta-percha carrier in single-rooted canals. J. Dent..

[32] Hammad M., Qualtrough A., Silikas N. (2008). Extended Setting Shrinkage Behavior of Endodontic Sealers. J. Endod..

[33] Shakya V.K., Gupta P., Tikku A.P., Pathak A.K., Chandra A., Yadav R.K., Bharti R., Singh R.K. (2016). An Invitro Evaluation of Antimicrobial Efficacy and Flow Characteristics for AH Plus, MTA Fillapex, CRCS and Gutta Flow 2 Root Canal Sealer. J. Clin. Diagn. Res..

[34] Alhashimi R.A., Foxton R., Romeed S., Deb S. (2014). An In Vitro Assessment of Gutta-Percha Coating of New Carrier-Based Root Canal Fillings. Sci. World J..

[35] Savariz A., Gonzalez-Rodriguez M., Ferrer-Luque C.M. (2010). Long-term sealing ability of GuttaFlow versus Ah Plus using different obturation techniques. Med. Oral. Patol. Oral. Cir. Bucal..

[36] Tzanetakis G.N., Kakavetsos V.D., Kontakiotis E.G. (2010). Impact of smear layer on sealing property of root canal obturation using 3 different techniques and sealers. Part I. Oral Surg. Oral Med. Oral. Pathol. Oral. Radiol. Endodontol..

[37] Al-Kahtani A.M. (2013). Carrier-based Root Canal Filling Materials: A Literature Review. J. Contemp. Dent. Pract..

[38] Kumar S., Deshpande S.J., Rao A.S. (2011). Comparison of apical sealing and periapical extrusion of the ThermaFil obturation technique with and without MTA as an apical barrier: An in vitro study. Indian J. Dent. Res..

[39] Vittoria G., Pantaleo G., Blasi A., Spagnuolo G., Iandolo A., Amato M. (2018). Thermafil: A New Clinical Approach Due to New Dimensional Evaluations. Open Dent. J..

[40] Pirani C., Iacono F., Gatto M.R., Fitzgibbon R.M., Chersoni S., Shemesh H., Prati C. (2018). Outcome of secondary root canal treatment filled with Thermafil: A 5-year follow-up of retrospective cohort study. Clin. Oral. Investig..

[41] Weis M.V., Parashos P., Messer H.H. (2004). Effect of obturation technique on sealer cement thickness and dentinal tubule penetration. Int. Endod. J..

[42] Zhong X., Shen Y., Ma J., Chen W., Haapasalo M. (2019). Quality of Root Filling after Obturation with Gutta-percha and 3 Different Sealers of Minimally Instrumented Root canals of the Maxillary First Molar. J. Endod..

